# Arachidonic Acid Disrupts PKM2‐Linked Glycolysis‐Related Metabolism and Exacerbates PAN‐Induced Podocyte Injury

**DOI:** 10.1111/jcmm.71404

**Published:** 2026-10-11

**Authors:** Kun Nie, Xiaowei Gao, Minggang Liu, Danyu You, Mengjie Weng, Tingting Zheng, Jiaqun Lin, Siyi Rao, Yongjie Zhuo, Jianxin Wan, Liyan Yang

**Affiliations:** ^1^ Department of Nephrology, Blood Purification Research Center, The First Affiliated Hospital Fujian Medical University Fuzhou China; ^2^ Fujian Clinical Research Center for Metabolic Chronic Kidney Disease, The First Affiliated Hospital Fujian Medical University Fuzhou China; ^3^ Department of Nephrology, National Regional Medical Center, Binhai Campus of the First Affiliated Hospital Fujian Medical University Fuzhou China

**Keywords:** arachidonic acid, glycolysis‐related metabolism, minimal change disease, mitochondrial dysfunction, PKM2, podocyte injury

## Abstract

Minimal change disease (MCD) frequently relapses, and infection is one of the most common clinical triggers. Arachidonic acid (AA), an inflammatory lipid mediator released during tissue injury and infection, has been implicated in podocyte injury, but its contribution to metabolic dysfunction in MCD remains unclear. Here, we investigated whether AA aggravates PAN‐induced podocyte injury and examined the involvement of pyruvate kinase M2 (PKM2)‐associated metabolic dysfunction. In a puromycin aminonucleoside (PAN)‐induced rat model, AA exposure increased serum AA levels, aggravated proteinuria, reduced nephrin expression, promoted foot‐process effacement and exacerbated mitochondrial dysfunction. AA was also associated with reduced PKM1 and PKM2 abundance, increased PKM2 (Tyr105) phosphorylation, decreased pyruvate kinase activity and lower pyruvate, lactate and ATP levels. In cultured human podocytes, AA intensified PAN‐induced cytoskeletal disruption, mitochondrial dysfunction, oxidative stress and apoptosis. Pharmacological activation of PKM2 with TEPP‐46 partially restored PK‐associated metabolic activity, improved mitochondrial readouts and attenuated podocyte injury in vitro. These findings suggest that AA exacerbates PAN‐induced podocyte injury in association with PKM2‐associated metabolic dysfunction and mitochondrial stress.

## 1 Introduction

Podocytes are highly specialized epithelial cells that form an essential component of the glomerular filtration barrier [1, 2]. Podocyte injury is a shared pathophysiological feature of podocytopathies, including minimal change disease (MCD), focal segmental glomerulosclerosis (FSGS), and membranous nephropathy (MN) [3]. Immune, infectious, drug‐related, metabolic, environmental, and genetic factors can damage podocytes or promote podocyte loss [4]. Among these triggers, infection is the most common clinical factor associated with recurrent proteinuria in podocytopathies [5, 6]. In adults, early‐onset and frequently relapsing MCD is often associated with respiratory infection, and many patients develop edema and massive proteinuria after infectious episodes [7]. Although immunosuppressive and supportive therapies are often initially effective, relapse remains common [8, 9]. The mechanisms by which infection‐associated inflammatory signals aggravate podocyte injury therefore remain incompletely defined.

Arachidonic acid (AA) is an ω‐6 polyunsaturated fatty acid that is widely distributed in human tissues and stored mainly in esterified form within cell‐membrane phospholipids. Circulating free AA is usually low because it rapidly binds albumin, with reported concentrations of approximately 0.1 μmol/L [10, 11]. During infection or tissue injury, inflammatory cytokines can activate membrane phospholipases, releasing free AA from cellular membranes and increasing circulating AA levels [12, 13, 14]. In kidney disease, proteinuria and hypoalbuminemia may further reduce albumin‐binding capacity and increase the biologically available AA pool. AA metabolism has been linked to renal inflammation and tissue injury [15, 16, 17], and previous work from our group and others has shown that excessive AA can promote podocyte injury. However, whether AA amplifies podocyte damage through metabolic stress in MCD‐related injury remains unclear.

Energy metabolism is essential for maintaining podocyte structure and filtration‐barrier function. Glycolysis and mitochondrial oxidative metabolism provide ATP and support cytoskeletal remodelling, redox adaptation and cellular homeostasis. Disruption of these pathways can cause metabolic stress, increase oxidative injury and aggravate podocyte damage [18, 19, 20]. Impaired glycolysis‐related metabolism has been observed in injured podocytes, whereas enhancing glycolytic activity can alleviate proteinuria and improve mitochondrial function in experimental podocyte injury [21, 22, 23]. These findings suggest that metabolic remodelling is closely linked to podocyte vulnerability. PKM2, a rate‐limiting glycolytic enzyme isoform, can regulate terminal glycolytic output through changes in abundance, oligomeric state, enzymatic activity and phosphorylation status.

Here, we tested whether AA aggravates established PAN‐induced podocyte injury and whether this effect is associated with PKM2‐linked metabolic dysfunction. Because exogenous AA does not recapitulate infection‐triggered clinical relapse, we interpret the PAN+AA condition as an AA‐associated injury‐exacerbation model. We combined rat kidney tissue and cultured human podocytes to assess structural injury, mitochondrial stress and glycolysis‐related metabolic endpoints, and further tested whether PKM2 activation with TEPP‐46 could attenuate PAN+AA‐induced injury in vitro.

## 2 Methods

### 2.1 Animals and Reagents

All animal experiments were performed according to protocols approved by the Institutional Animal Care and Use Committee of Fujian Medical University (Approval No. IACUC FJMU 2022‐0636). Male SPF‐grade Sprague–Dawley rats (8 weeks old, 180–200 g) were housed under pathogen‐free conditions and randomly assigned to four groups (*n* = 6 per group): Control, PAN, PAN+AA, and AA. To induce podocyte injury resembling minimal change disease (MCD), rats in the PAN and PAN+AA groups received a single intraperitoneal injection of puromycin aminonucleoside (PAN; M6290, Abmole, USA) at 7 mg per 100 g body weight. Ten days after PAN injection, rats in the PAN+AA group received intraperitoneal arachidonic acid (AA; 10,931, Sigma, USA) at 3 mg/kg to evaluate whether AA exposure exacerbates established PAN‐induced podocyte injury. Rats in the AA group received a single intraperitoneal injection of AA at the same dose. This PAN+AA protocol was used as an AA‐associated injury‐exacerbation model and was not intended to reproduce the full clinical process of infection‐triggered MCD relapse. Twenty‐four‐hour urine samples were collected weekly using metabolic cages (Tecniplast, Italy) for biochemical analysis. All animals were euthanized 24 h after AA administration to the PAN+AA and AA groups, corresponding to Day 11 after PAN injection in the PAN and PAN+AA groups. Before euthanasia, rats were anaesthetised by intraperitoneal injection of 10% chloral hydrate (0.3 mL/100 g). Blood was collected by abdominal aortic puncture, after which the animals were euthanized and both kidneys were harvested. After removal of the renal capsule, kidneys were sagittally sectioned and either fixed in 4% paraformaldehyde or snap‐frozen in liquid nitrogen for subsequent analyses.

### 2.2 Renal Function Assays and Serum AA Measurement

Blood samples were centrifuged at 3000 rpm for 10 min at 4°C to obtain serum. Serum creatinine, blood urea nitrogen, and 24‐h urinary albumin levels were measured using a fully automated biochemical analyser (Roche, Switzerland). Serum arachidonic acid (AA) levels were measured using a commercially available enzyme‐linked immunosorbent assay (ELISA) kit (Elabscience, catalogue no. E‐EL‐0051) according to the manufacturer's instructions. Briefly, serum samples from the Control, PAN, PAN+AA, and AA groups were thawed on ice and analysed in accordance with the kit protocol. Absorbance was measured using a microplate reader at the recommended wavelength, and AA concentrations were calculated from the standard curve. Serum AA levels were expressed as ng/mL.

### 2.3 Histological Analysis

Kidney tissues were fixed in 4% paraformaldehyde and embedded in paraffin. Sections (4 μm) were prepared and stained according to standard protocols for haematoxylin and eosin, Masson's trichrome, and periodic acid‐Schiff staining.

### 2.4 Transmission Electron Microscopy (TEM)

Kidney ultrastructure was examined by transmission electron microscopy. Briefly, rats were perfusion‐fixed with 2.5% glutaraldehyde in 0.1 M phosphate‐buffered saline (PBS, pH 7.4). Renal cortical tissues were post‐fixed in 1% osmium tetroxide, dehydrated through a graded ethanol series, and embedded in Polybed 812 epoxy resin following infiltration with a 1:1 mixture of propylene oxide and resin. Ultrathin sections (100 nm) were prepared using a Leica UC6 ultramicrotome (Leica Microsystems, Germany), double‐stained with 2% uranyl acetate and 1% lead citrate, and examined using a transmission electron microscope. Cultured podocytes used for TEM analysis were fixed in 2.5% glutaraldehyde and subsequently processed using the same post‐fixation, dehydration, embedding, ultrathin sectioning, staining, and imaging procedures.

### 2.5 Cell Culture and Intervention

The conditionally immortalized human podocyte cell line was kindly provided by Dr. Moin A. Saleem (University of Bristol, UK). Podocytes were propagated at 33°C in RPMI‐1640 medium supplemented with 10% fetal bovine serum and insulin‐transferrin‐selenium. To induce differentiation, confluent cells were transferred to medium containing 10% fetal bovine serum without insulin‐transferrin‐selenium and cultured at 37°C for 10–14 days. Differentiated podocytes were serum‐starved in medium containing 2% fetal bovine serum for 24 h before experiments. For interventions, stock solutions of PAN (250 mM) and AA (5 × 10^−5^ M) were prepared in culture medium. Differentiated podocytes were treated for 24 h with PAN (250 μmol/L), AA (5 × 10^−7^ M), or both in medium containing 10% fetal bovine serum. TEPP‐46 (ML‐265), a selective PKM2 activator, was dissolved in DMSO to prepare a 10 mM stock solution and diluted in complete medium to the required working concentration. The final DMSO concentration was kept below 0.1% in all groups.

### 2.6 Immunofluorescence

Renal tissue sections and cell slides were thoroughly washed with PBS, permeabilized with PBS containing 0.3% Triton X‐100, and blocked with 5% BSA for 30 min. Samples were incubated with primary antibodies overnight at 4°C. Depending on the experiment, the primary antibodies included: guinea pig anti‐Nephrin (1:200, PROGEN, Germany), rabbit anti‐PKM1 (1:200, Abcam, USA), rabbit anti‐PKM2 (1:100, Proteintech), rabbit anti‐p‐PKM2 (1:100, Proteintech), and rabbit anti‐TOM20 (1:100, CST). After primary antibody incubation, sections were washed with PBS and incubated with Alexa Fluor 488‐or Alexa Fluor 594‐conjugated goat anti‐guinea pig or goat anti‐rabbit secondary antibodies for 1 h at room temperature, followed by counterstaining with DAPI for 10 min at room temperature. For immunofluorescence quantification, images were acquired using identical microscope settings across groups. The nephrin‐positive area within each glomerulus was defined as the podocyte region of interest. TOM20 fluorescence intensity was quantified within the nephrin‐positive area after background subtraction. The same thresholding, region‐of‐interest selection, and background‐correction strategy was applied consistently for nephrin‐associated quantification of PKM1, PKM2, phospho‐PKM2, and TOM20 staining.

### 2.7 Cytoskeleton

Cells were fixed with 4% paraformaldehyde for 15 min, permeabilized with PBS containing 0.3% Triton X‐100, blocked with 5% BSA for 30 min, and stained with 500 nM Alexa Fluor 488 phalloidin (Invitrogen) for 30 min and 1 × Hoechst for 15 min. Phalloidin‐labelled F‐actin (green) and Hoechst stain cellular DNA (blue). Cytoskeletal organization was visualized using merged two‐channel images.

### 2.8 Mitochondrial Morphology and Mitochondrial Membrane Potential

MitoTracker Red CMXRos (M7512, Invitrogen) was added to culture medium at a final concentration of 100 nM to visualize mitochondrial network morphology, and cells were incubated at 37°C for 30 min. After incubation, cells were gently washed and fixed. Mitochondrial membrane potential was assessed using the potential‐dependent accumulation of JC‐1 (MA0338, Meilunbio, China). Cells were washed twice with PBS and incubated with JC‐1 for 30 min at 37°C in the dark. Fluorescence signals were quantified by confocal microscopy (ZEISS LSM800) or flow cytometry (FACS).

### 2.9 Podocyte ATP Measurement

Podocytes were seeded in 12‐well plates at a density of 2 × 10^4^ cells per well. After differentiation and serum starvation, cells were treated with PAN (250 μmol/L), AA (5 × 10^−7^ M), PAN plus AA, or the corresponding vehicle for 24 h. For the TEPP‐46 rescue experiment, cells were treated with PAN plus AA in the presence or absence of TEPP‐46 (10 μmol/L) for 24 h. ATP levels were measured using an ATP Bioluminescence Assay Kit (S0027, Beyotime, China) according to the manufacturer's instructions. Luminescence was normalised to the total protein concentration.

### 2.10 Measurement of Pyruvate, Lactate, and Pyruvate Kinase Activity

Lactate concentrations in cell extracts were measured using a Lactate Colorimetric Assay Kit (Cat. No. K044‐M, Elabscience, China) according to the manufacturer's instructions. Pyruvate concentrations were determined using a Pyruvate Assay Kit (Cat. No. BC205, Solarbio, China) according to the manufacturer's protocol. To further assess glycolysis‐related enzymatic function, total pyruvate kinase (PK) activity was measured in rat kidney tissue and cultured podocyte lysates using a commercial PK activity assay kit (E‐BC‐K611M, Elabscience, China) according to the manufacturer's instructions. Briefly, frozen kidney tissue samples and cultured podocytes were lysed or homogenized in the recommended extraction buffer and centrifuged to remove insoluble material. The supernatant was collected for PK activity analysis, and protein concentration was determined for normalization. PK activity was calculated according to the standard curve or formula provided by the kit and expressed as U/g protein.

### 2.11 Reactive Oxygen Species (ROS) Fluorescence and Flow Cytometry Analysis

Intracellular ROS levels were assessed using DCFH‐DA staining. For fluorescence microscopy, DCFH‐DA was diluted 1:1000 in serum‐free culture medium to a final concentration of 10 μmol/L. After the culture medium was removed, diluted DCFH‐DA solution was added to cover the cell monolayer, and cells were incubated at 37°C for 20 min in the dark. Cells were then washed to remove excess probe, nuclei were counterstained with DAPI, and fluorescence images were acquired using a fluorescence microscope. For flow‐cytometry analysis, cells were collected and resuspended in diluted DCFH‐DA solution at a density of 10^6^–10^7^ cells/mL. After incubation at 37°C for 20 min in the dark, cells were washed and analysed using a flow cytometer. ROS levels were quantified based on DCF fluorescence intensity.

### 2.12 TUNEL Assay

Cells were fixed with 4% paraformaldehyde for 10 min. Deparaffinized sections and fixed cell slides were incubated with 20 μg/mL DNase‐free proteinase K for 30 min at room temperature and then with TdT reaction mix for 60 min at 37°C in the dark. Nuclei were counterstained with DAPI, and images were acquired by fluorescence microscopy. Apoptotic cells were quantified by counting TUNEL‐positive cells in three randomly selected fields per sample.

### 2.13 Western Blotting

Proteins were extracted from kidney tissues and podocytes using RIPA lysis buffer containing protease and phosphatase inhibitor cocktails (Beyotime, Shanghai, China) according to the manufacturer's protocol. Protein concentrations were determined using a BCA protein assay kit (Beyotime). Proteins were separated by 10% SDS‐PAGE, transferred onto polyvinylidene difluoride membranes (Millipore, Billerica, MA, USA), and blocked with 5% non‐fat milk for 2 h at room temperature. Membranes were incubated overnight at 4°C with primary antibodies against β‐actin (1:2000, Sigma‐Aldrich, MO, USA), nephrin (1:500, PROGEN), PKM1 (1:1000, Abcam), PKM2 (1:1000, Proteintech), phospho‐PKM2 (Tyr105 1:1000, Proteintech), β‐actin (1:1000, CST), and TOMM20 (1:1000, CST). Immunoreactive proteins were visualized using a chemiluminescence imaging system (Tanon, Shanghai, China).

### 2.14 Image Quantification

Image quantification was performed using ImageJ/Fiji and ZEN software, as appropriate. For TEM analysis of podocyte ultrastructure, glomerular basement membrane (GBM) thickness, foot‐process width, and the number of foot processes per μm GBM were quantified from glomerular capillary loops. For mitochondrial TEM analysis, mean mitochondrial area, cristae‐damage rate, and mitochondrial damage score were quantified. For F‐actin staining, cytoskeletal organization was assessed by quantifying F‐actin fluorescence intensity. For immunofluorescence images, mean fluorescence intensity was quantified using ZEN software. For glomerular staining, fluorescence intensity was quantified within nephrin‐positive regions where applicable. Images from the same experiment were acquired using identical microscope settings, and the same analysis parameters were applied across all groups.

### 2.15 Statistical Analysis

Statistical analyses were performed using GraphPad Prism 10. Data are presented as mean ± SD. For animal experiments, n represents biological replicates, with each rat considered one independent experimental unit. For in vitro podocyte experiments, n represents independent biological replicates from separate cell‐culture experiments unless otherwise stated. Technical replicates within an assay were averaged before statistical analysis and were not treated as independent biological replicates. Sample sizes for each assay are indicated in the corresponding figure legends. Comparisons among three or more independent groups were analysed using ordinary one‐way ANOVA followed by Bonferroni's multiple‐comparisons test. Two‐group comparisons were analysed using unpaired Student's *t*‐test. Normality was assessed using the Shapiro–Wilk test, and homogeneity of variance was assessed using the Brown‐Forsythe test. *p* < 0.05 was considered statistically significant.

## 3 Results

### 3.1 AA Exacerbated Glomerular Podocyte Injury in PAN‐Induced Rats

MCD is a glomerular disorder primarily characterized by podocyte injury. Arachidonic acid (AA), an inflammatory lipid mediator released during inflammatory or infectious stress, may aggravate podocyte injury and proteinuria. In this study, we established a PAN‐induced podocyte injury model and administered AA to determine whether AA exposure exacerbates established PAN‐induced renal injury. The PAN+AA protocol was interpreted as an AA‐associated injury‐exacerbation model rather than a complete clinical relapse model. To evaluate systemic AA exposure, serum AA levels were measured by ELISA. Serum AA was increased in PAN‐treated rats compared with Controls (8.242 vs. 4.480 ng/mL, adjusted *p* = 0.0410) and was further increased in PAN+AA rats (11.96 ng/mL; Control vs. PAN+AA, adjusted *p* < 0.0001; PAN vs. PAN+AA, adjusted *p* = 0.0441). Serum AA was also higher in PAN+AA rats than in AA‐only rats (11.96 vs. 5.864 ng/mL, adjusted *p* = 0.0005). The PAN+AA group showed more severe renal injury than the PAN group, as indicated by increased urinary albumin excretion and elevated serum AA levels (Figure 1A). Consistent with the typical light‐microscopic features of MCD injury, haematoxylin and eosin, Masson's trichrome, and periodic acid‐Schiff staining showed no prominent glomerular sclerosis, inflammatory infiltration, or interstitial fibrosis among the groups under light microscopy (Figure 1B). Therefore, we further examined podocyte ultrastructure by transmission electron microscopy. TEM revealed podocyte ultrastructural damage in the PAN group, which was further aggravated in the PAN+AA group (Figure 1C). Quantitative analysis of transmission electron microscopy images confirmed these ultrastructural changes. GBM thickness increased from 155.0 nm in Control rats to 226.7 nm in PAN‐treated rats and 273.7 nm in PAN+AA‐treated rats, with a significant difference between PAN and PAN+AA groups (*p* = 0.0023). Foot‐process width increased from 0.4233 μm in Control rats to 0.7267 μm in PAN‐treated rats and 1.003 μm in PAN+AA‐treated rats (PAN vs. PAN+AA, *p* = 0.0009). In contrast, the number of foot processes per μm GBM decreased from 2.043 in Control rats to 1.157 in PAN‐treated rats and 0.8067 in PAN+AA‐treated rats (PAN vs. PAN+AA, *p* = 0.0108). Nephrin, a slit‐diaphragm protein essential for glomerular filtration‐barrier function, was reduced in both AA‐ and PAN‐treated rats compared with Control rats. Immunofluorescence and Western blot analyses of kidney tissue lysates showed a further decrease in nephrin protein expression in the PAN+AA group (Figure 1D,E). These findings indicate that AA aggravates PAN‐induced podocyte injury and filtration‐barrier dysfunction.

**FIGURE 1:**
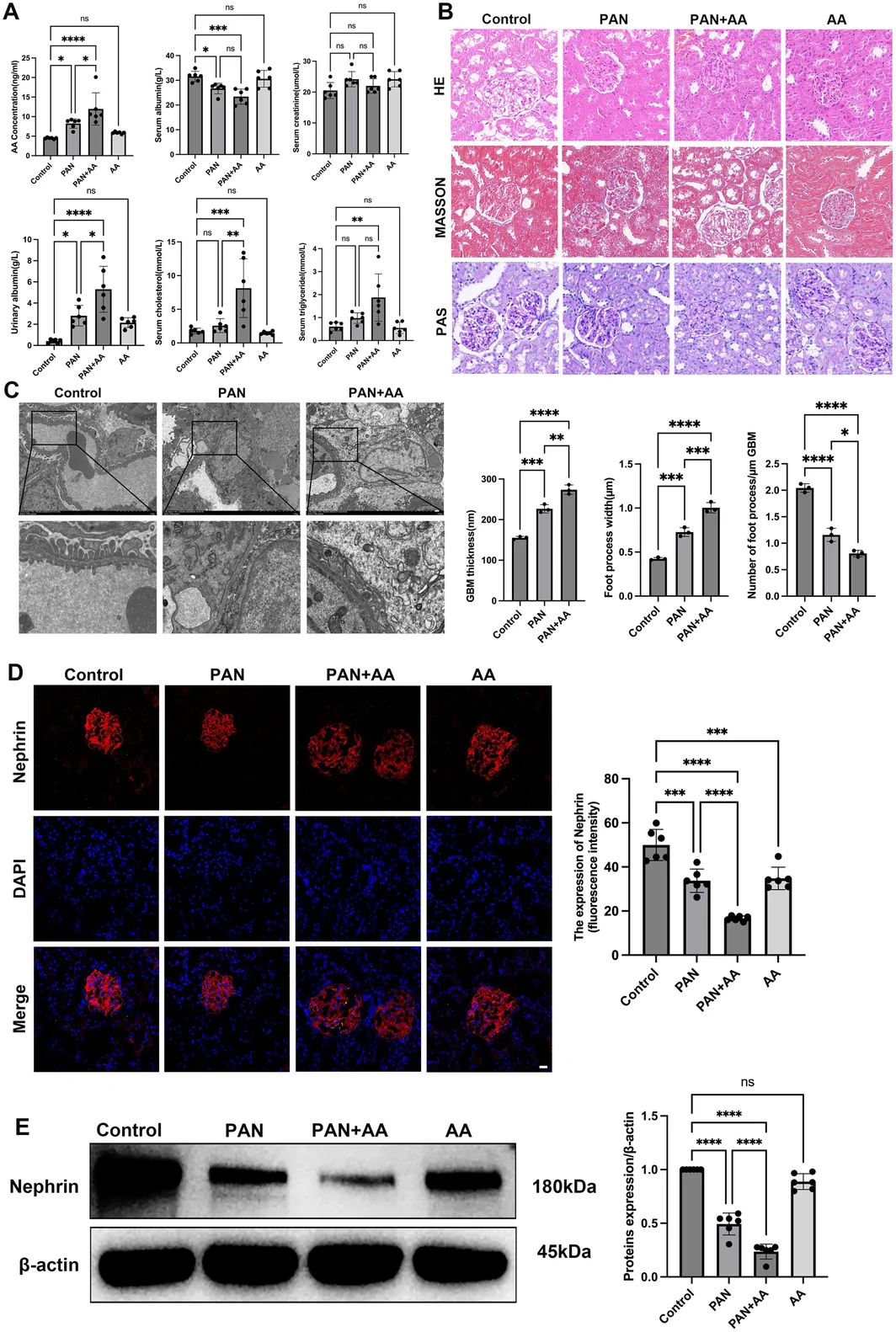
AA exacerbated podocyte injury in PAN‐induced rats. Eight‐week‐old male Sprague–Dawley rats (180–200 g) were subjected to puromycin aminonucleoside (PAN)‐induced podocyte injury by a single intraperitoneal injection of PAN (7 mg/100 g body weight). Ten days after PAN injection, rats in the PAN+AA group received arachidonic acid (AA, 3 mg/kg, i.p.) to evaluate AA‐associated exacerbation of established PAN‐induced injury. (A) Serum AA levels were measured by ELISA. Serum albumin, serum creatinine, 24‐h urinary albumin, serum cholesterol and serum triglyceride levels were measured as indicators of renal function and metabolic status (*n* = 6). (B) Representative haematoxylin and eosin (H&E), Masson's trichrome, and periodic acid‐Schiff (PAS) staining of kidney sections (*n* = 6). Scale bar, 20 μm. (C) Representative transmission electron microscopy images of podocyte foot processes and quantitative analysis of glomerular basement membrane (GBM) thickness, foot‐process width, and the number of foot processes per μm GBM (*n* = 3). Scale bar, 5.0 μm. (D) Representative immunofluorescence staining of nephrin in glomeruli. Nephrin is shown in red and nuclei are counterstained with DAPI in blue (*n* = 6). Scale bar, 20 μm. (E) Representative Western blot analysis and quantification of nephrin protein expression in renal tissue lysates (*n* = 6). Data are expressed as the mean ± SD and analysed by one‐way ANOVA with Bonferroni correction. *p < 0.05, **p < 0.01, ***p < 0.001, ****p < 0.0001; ns, not significant. AA, arachidonic acid; GBM, glomerular basement membrane; i.p., intraperitoneal; MCD, minimal change disease; PAN, puromycin aminonucleoside.

### 3.2 AA Was Associated With Reduced PK Activity, Altered Glycolysis‐Related Endpoints and Aggravated Mitochondrial Dysfunction

Transmission electron microscopy showed marked mitochondrial injury in podocytes from PAN+AA‐treated rats. Compared with PAN alone, PAN+AA increased mitochondrial swelling and size heterogeneity, with extensive matrix rarefaction and fragmented or dissolved cristae (Figure 2A). Quantitative analysis further showed a larger mean mitochondrial area, a higher cristae‐damage rate and an increased mitochondrial damage score in the PAN+AA group (Figure 2A). These ultrastructural changes indicated that AA aggravated PAN‐associated mitochondrial damage in vivo. We next assessed TOM20, a mitochondrial outer‐membrane protein, together with the podocyte marker nephrin. Immunofluorescence analysis showed that TOM20 signal within nephrin‐positive glomerular regions was reduced after PAN treatment and was further decreased in the PAN+AA group (Figure 2B). Consistently, Western blotting of renal tissue lysates confirmed a further reduction in TOM20 protein abundance in PAN+AA‐treated rats compared with PAN‐treated rats (Figure 2C). Together, the TEM, immunofluorescence and Western blot findings showed more severe mitochondrial structural injury and reduced TOM20 abundance after AA exposure in the PAN‐induced injury setting. Because mitochondrial injury can be linked to altered energy metabolism, we next examined glycolysis‐related PKM isoforms in renal tissue from PAN‐induced rats. Immunofluorescence staining showed that PKM1 signal within nephrin‐positive glomerular regions was reduced after PAN treatment and was further decreased in the PAN+AA group (Figure 3A). Western blotting of renal cortex lysates showed a similar reduction in total PKM1 protein abundance (Figure 3B). PKM2 showed the same pattern: PKM2 fluorescence in nephrin‐positive regions was lower in PAN‐treated rats and was further reduced after AA administration (Figure 3C). Consistently, renal cortical PKM2 protein levels were decreased in the PAN+AA group compared with the PAN group (Figure 3D). In contrast, phospho‐PKM2 staining and renal cortical phospho‐PKM2 protein abundance were increased in PAN+AA‐treated rats (Figure 3E,F). These results indicate that AA exposure was associated with reduced PKM1 and PKM2 abundance and increased PKM2 phosphorylation in PAN‐induced renal injury.

**FIGURE 2:**
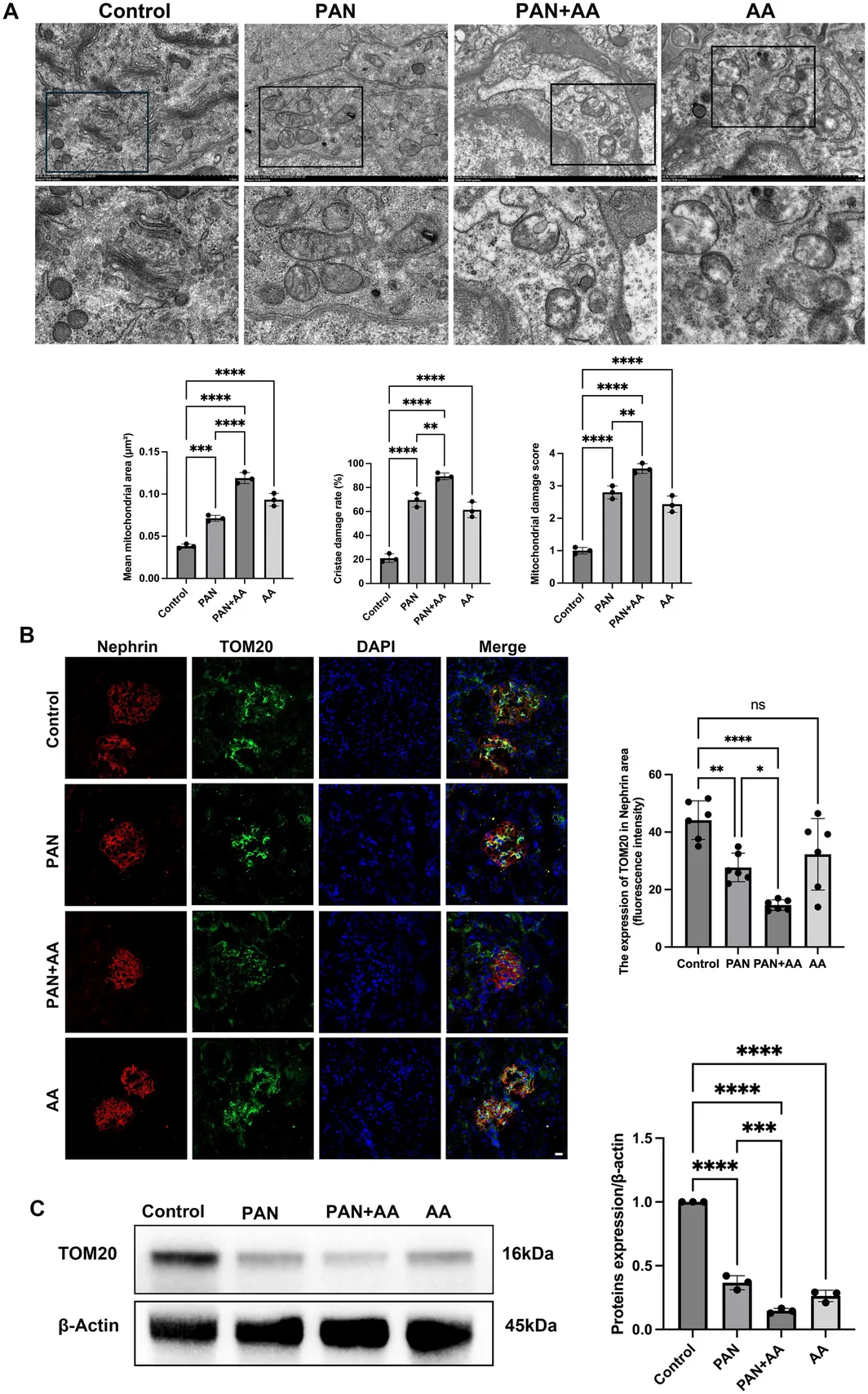
AA aggravated mitochondrial structural injury in podocytes from PAN‐induced rats. (A) Representative transmission electron microscopy images of podocyte mitochondria and quantification of mean mitochondrial area, cristae‐damage rate and mitochondrial damage score (*n* = 3). Scale bar, 1.0 μm. (B) Representative immunofluorescence images showing Nephrin (red), TOM20 (green), DAPI (blue) and merged signals in glomeruli, with quantification of TOM20 fluorescence within nephrin‐positive regions (*n* = 6). Scale bar, 20 μm. (C) Western blot analysis and quantification of TOM20 protein expression in renal tissue lysates (*n* = 3). Data are expressed as the mean ± SD and analysed by one‐way ANOVA with Bonferroni correction. *p < 0.05, **p < 0.01, ***p < 0.001, ****p < 0.0001; ns, not significant.

**FIGURE 3:**
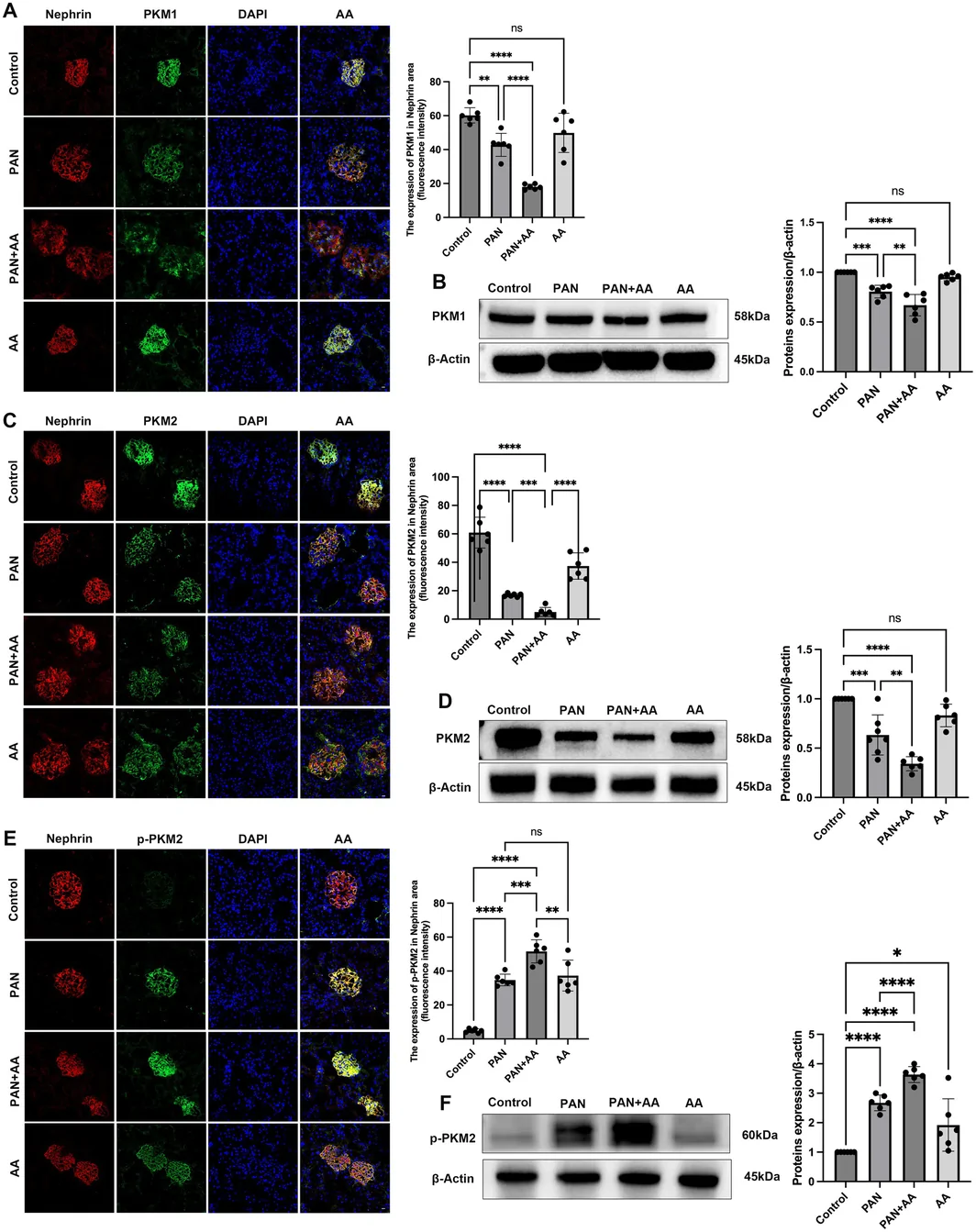
AA altered PKM1, PKM2 and phospho‐PKM2 expression in PAN‐induced rat kidneys. (A) Representative immunofluorescence images of Nephrin (red), PKM1 (green), DAPI (blue) and merged signals in glomeruli, with quantification of PKM1 fluorescence within nephrin‐positive regions (*n* = 6). Scale bar, 20 μm. (B) Western blot analysis and quantification of PKM1 protein expression in renal cortex lysates (*n* = 6). (C) Representative immunofluorescence images of Nephrin (red), PKM2 (green), DAPI (blue) and merged signals in glomeruli, with quantification of PKM2 fluorescence within nephrin‐positive regions (*n* = 6). Scale bar, 20 μm. (D) Western blot analysis and quantification of PKM2 protein expression in renal cortex lysates (*n* = 6). (E) Representative immunofluorescence images of Nephrin (red), phospho‐PKM2 (green), DAPI (blue) and merged signals in glomeruli, with quantification of phospho‐PKM2 fluorescence within nephrin‐positive regions (*n* = 6). Scale bar, 20 μm. (F) Western blot analysis and quantification of phospho‐PKM2 protein expression in renal cortex lysates (*n* = 6). Data are expressed as the mean ± SD and analysed by one‐way ANOVA with Bonferroni correction. *p < 0.05, **p < 0.01, ***p < 0.001, ****p < 0.0001; ns, not significant.

Having observed altered PKM expression and phosphorylation, we next examined whether these changes were accompanied by reduced PK enzymatic activity and altered energy‐related metabolites. Total PK activity was reduced in PAN‐treated renal tissue and declined further in the PAN+AA group (Figure 4A). Consistently, renal pyruvate and lactate levels were lower after PAN treatment and were further decreased after AA administration (Figure 4B,C). ATP content followed the same pattern, with the lowest levels detected in the PAN+AA group (Figure 4D). These findings indicate that AA exposure was associated with reduced PK enzymatic activity and impaired glycolysis‐related energy metabolism in PAN‐induced renal injury.

**FIGURE 4:**
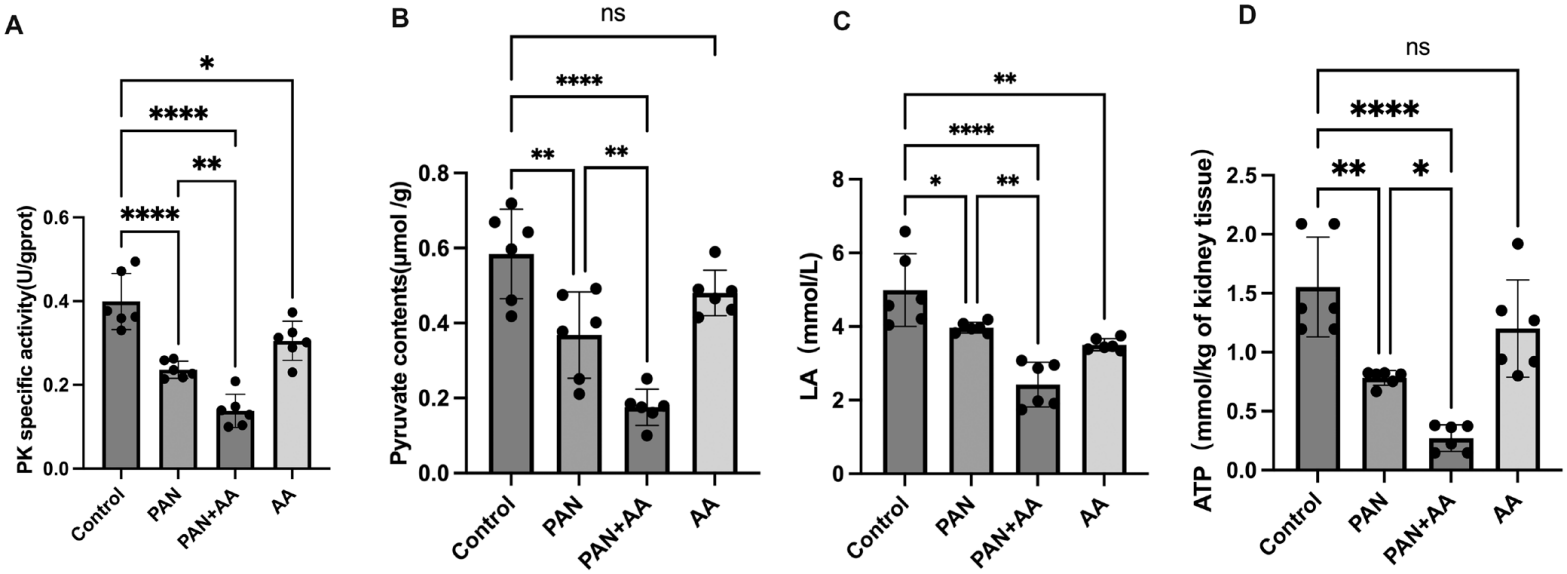
AA reduced PK activity and energy‐related metabolite levels in PAN‐induced rat kidneys. (A) Total pyruvate kinase (PK) activity in renal tissue (*n* = 6). (B) Pyruvate levels in renal tissue (*n* = 6). (C) Lactate (LA) levels in renal tissue (*n* = 6). (D) ATP content in renal tissue (*n* = 6). Data are expressed as the mean ± SD and analysed by one‐way ANOVA with Bonferroni correction. *p < 0.05, **p < 0.01, ***p < 0.001, ****p < 0.0001; ns, not significant.

### 3.3 AA Exacerbated PAN‐Induced Podocyte Injury In Vitro

To determine whether AA directly aggravates PAN‐induced podocyte injury, differentiated human podocytes were exposed to PAN, AA or PAN+AA in vitro. Phalloidin staining showed well‐organized F‐actin stress fibres in control podocytes, whereas PAN treatment disrupted cytoskeletal organization and reduced F‐actin intensity (Figure 5A). This effect was more pronounced in the PAN+AA group, which showed further loss of organized actin fibres and the lowest F‐actin mean intensity (Figure 5A). Consistently, Western blotting showed that nephrin protein expression was reduced after PAN treatment and was further decreased after combined PAN+AA exposure (Figure 5B). These findings indicate that AA aggravated PAN‐induced podocyte cytoskeletal injury and loss of nephrin expression in vitro.

**FIGURE 5:**
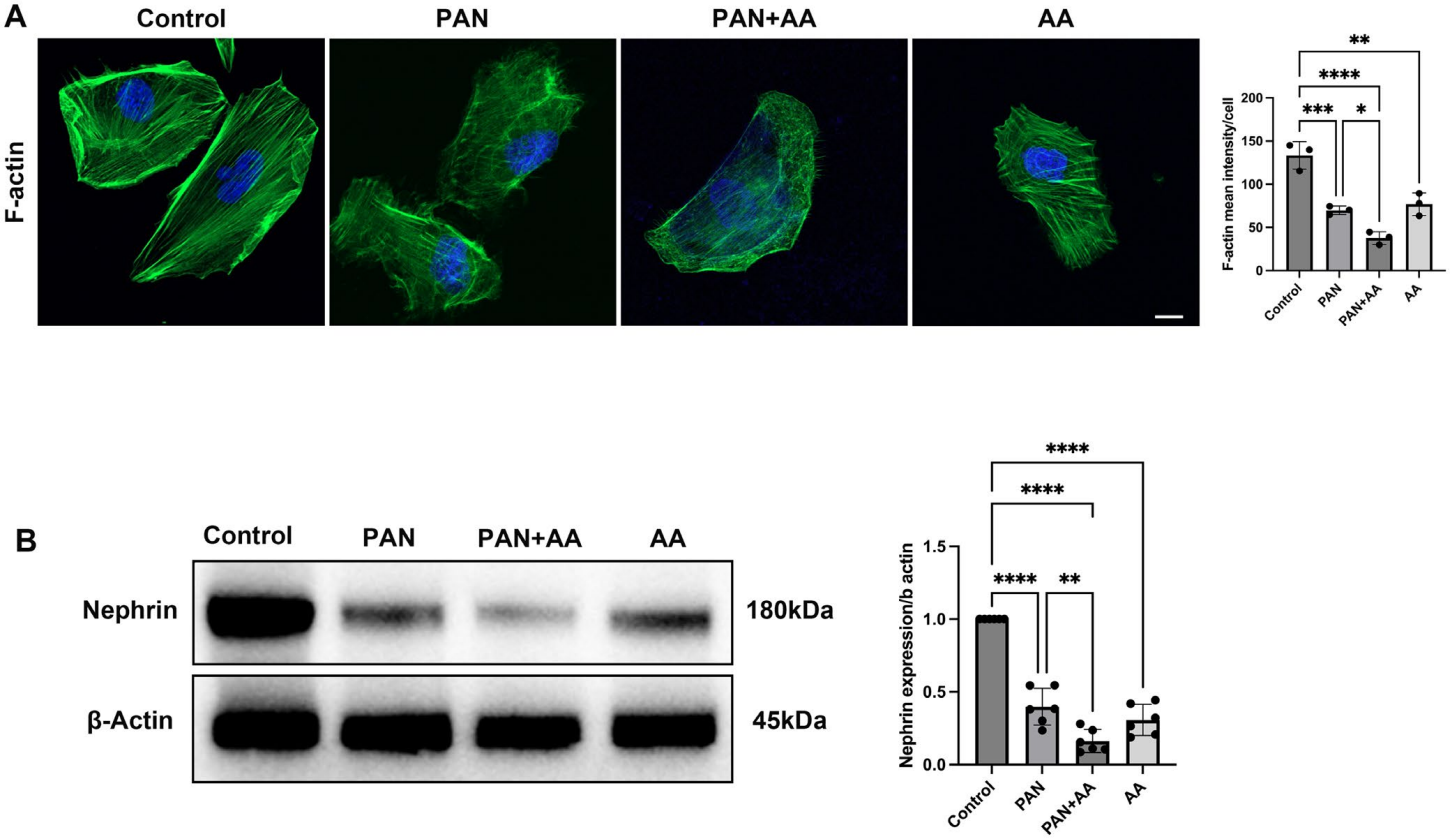
Aa aggravated PAN‐induced podocyte injury in vitro. Control podocytes were treated with PBS. PAN‐treated podocytes received 250 μM PAN for 24 h. PAN+AA‐treated podocytes received PAN (250 μM) and AA (5 × 10^−7^ M) for 24 h. AA‐treated podocytes received AA (5 × 10^−7^ M) for 24 h. (A) Representative phalloidin staining images of F‐Actin (green) and nuclei (blue), with quantification of F‐Actin mean intensity per cell (*n* = 3). Scale bar, 20 μm. (B) Western blot analysis and quantification of nephrin protein expression in cultured podocytes (*n* = 6). Data are expressed as the mean ± SD and analysed by one‐way ANOVA with Bonferroni correction. *p < 0.05, **p < 0.01, ***p < 0.001, ****p < 0.0001; ns, not significant.

### 3.4 AA Altered PKM‐Associated Glycolytic Metabolism and Aggravated Mitochondrial Dysfunction in PAN‐Induced Podocytes

After confirming that AA aggravated PAN‐induced podocyte injury, we examined whether this effect was accompanied by altered PKM‐associated metabolic regulation. Immunofluorescence analysis showed that PKM1 and PKM2 signals were reduced in PAN‐treated podocytes and were further decreased after combined PAN+AA exposure (Figure 6A,B). By contrast, phospho‐PKM2 staining was increased in the PAN+AA group (Figure 6C). Western blotting confirmed lower PKM1 and PKM2 protein levels and higher phospho‐PKM2 abundance in PAN+AA‐treated podocytes (Figure 6D–F). Consistent with these changes, PK activity was reduced after PAN treatment and was further decreased in the PAN+AA group (Figure 6G). Lactate, pyruvate and ATP levels showed a similar reduction after combined PAN+AA exposure (Figure 6G). These data indicate that AA was associated with reduced PK enzymatic activity and impaired glycolysis‐related energy metabolism in PAN‐induced podocytes.

**FIGURE 6:**
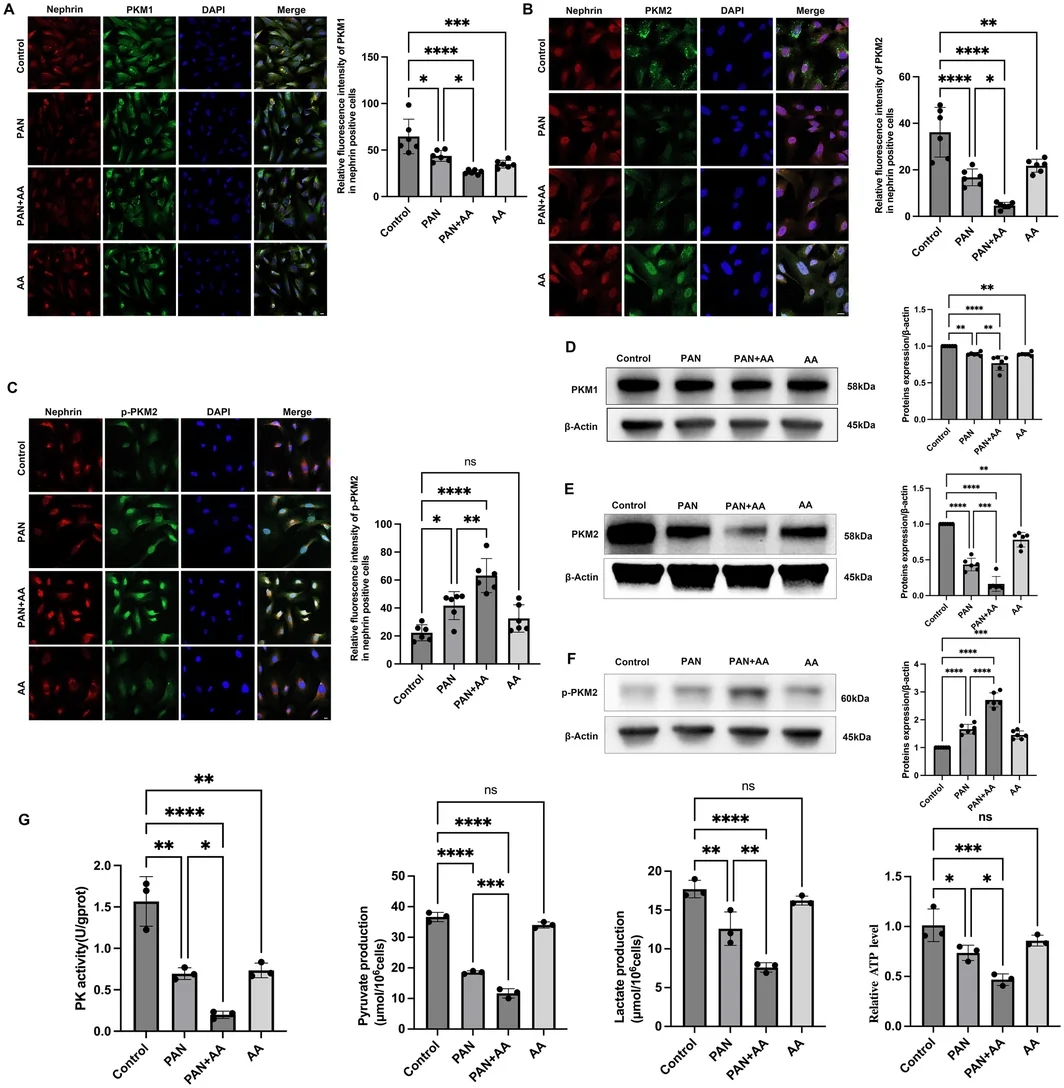
AA altered PKM expression, PK activity and glycolysis‐related metabolites in PAN‐induced podocytes. Control podocytes were treated with PBS. PAN‐treated podocytes were exposed to PAN (250 μM) for 24 h. PAN+AA‐treated podocytes were exposed to PAN (250 μM) and AA (5 × 10^−7^ M) for 24 h. AA‐treated podocytes were exposed to AA (5 × 10^−7^ M) for 24 h. (A‐C) Representative immunofluorescence images and quantification of PKM1, PKM2 and phospho‐PKM2 in podocytes. Nephrin is shown in red, PKM1, PKM2 or phospho‐PKM2 in green, and DAPI in blue (*n* = 6). Scale bar, 20 μm. (D‐F) Western blot analysis and quantification of PKM1, PKM2 and phospho‐PKM2 protein expression (*n* = 6). (G) PK activity, lactate production, pyruvate production and ATP levels in cultured podocytes (*n* = 3). Data are expressed as the mean ± SD and analysed by one‐way ANOVA with Bonferroni correction. *p < 0.05, **p < 0.01, ***p < 0.001, ****p < 0.0001; ns, not significant.

We next assessed mitochondrial structure and function in the same in vitro model. Transmission electron microscopy showed mitochondrial swelling, increased mitochondrial area, cristae disruption and a higher mitochondrial damage score in PAN‐treated podocytes, with more severe changes in the PAN+AA group (Figure 7A). JC‐1 staining showed a reduced aggregate‐to‐monomer ratio after PAN treatment, indicating loss of mitochondrial membrane potential, and this reduction was further enhanced by AA (Figure 7B). TOM20 immunofluorescence and Western blotting showed reduced mitochondrial marker expression in PAN‐treated podocytes, with the lowest levels in the PAN+AA group (Figure 7C,D). MitoSOX staining further showed that AA increased mitochondrial ROS accumulation under PAN‐induced conditions (Figure 7E). Together, these findings show that AA aggravated PAN‐induced mitochondrial structural damage, loss of mitochondrial membrane potential and mitochondrial oxidative stress in podocytes.

**FIGURE 7:**
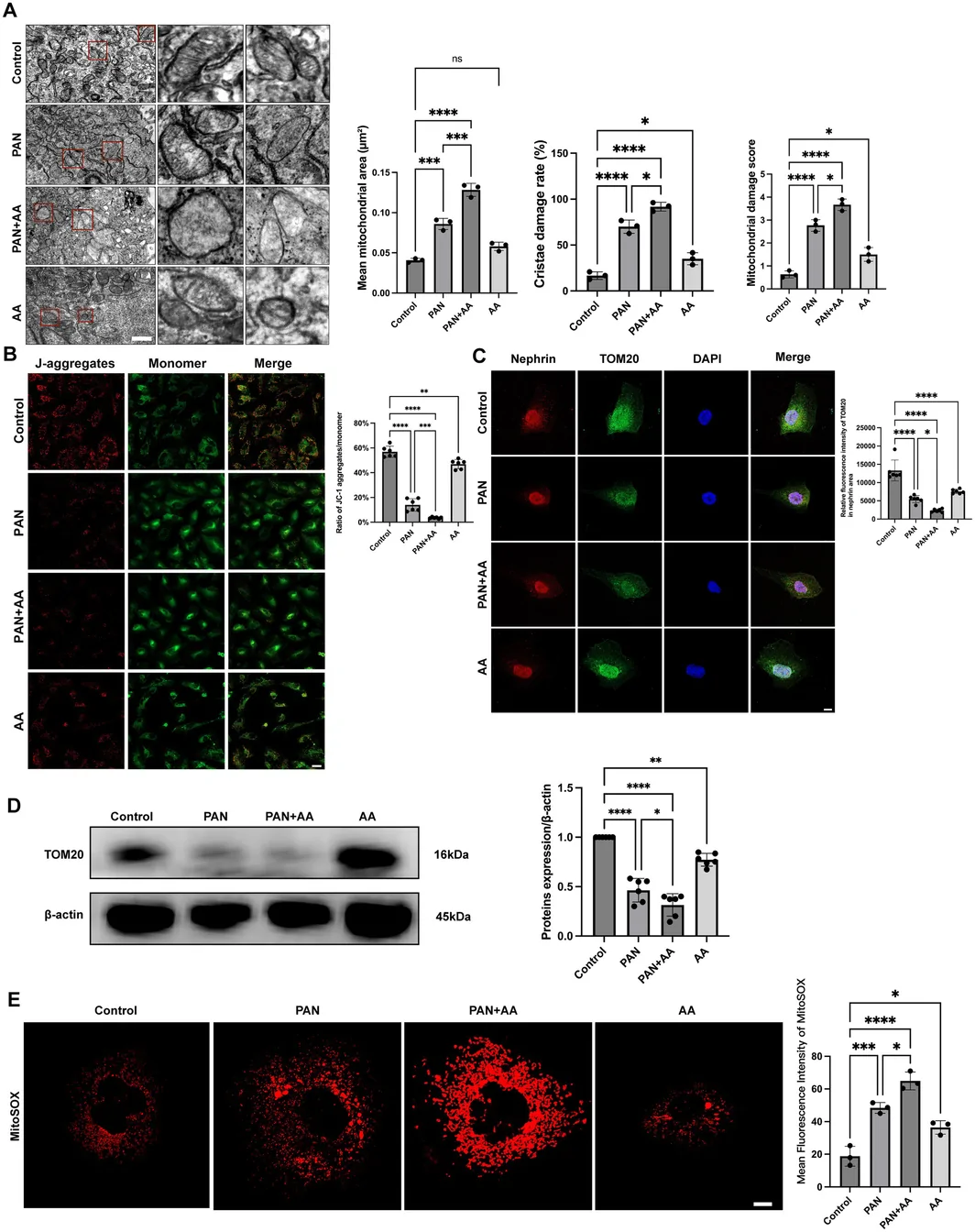
AA aggravated mitochondrial dysfunction and oxidative stress in PAN‐induced podocytes. (A) Representative transmission electron microscopy images and quantification of mean mitochondrial area, cristae‐damage rate and mitochondrial damage score (*n* = 3). Scale bar, 2.0 μm. (B) JC‐1 staining and quantification of the aggregate‐to‐monomer fluorescence ratio (*n* = 6). Scale bar, 20 μm. (C) Representative immunofluorescence images and quantification of TOM20 in nephrin‐positive podocytes. Nephrin is shown in red, TOM20 in green and DAPI in blue (*n* = 6). Scale bar, 10 μm. (D) Western blot analysis and quantification of TOM20 protein expression (*n* = 6). (E) Representative MitoSOX staining images and quantification of mitochondrial ROS levels (*n* = 3). Scale bar, 20 μm. Data are expressed as the mean ± SD and analysed by one‐way ANOVA with Bonferroni correction. *p < 0.05, **p < 0.01, ***p < 0.001, ****p < 0.0001; ns, not significant.

Given the increase in mitochondrial ROS, we next examined whether AA also enhanced cellular oxidative stress and apoptosis in PAN‐treated podocytes. TUNEL staining showed that PAN increased the proportion of apoptotic podocytes, and this increase was further enhanced by combined PAN+AA exposure (Figure 8A). DCFH‐DA staining showed higher intracellular ROS levels in PAN‐treated podocytes, with a further increase in the PAN+AA group (Figure 8B). Flow‐cytometric analysis showed the same pattern of increased DCFH fluorescence after PAN+AA treatment (Figure 8B). These results indicate that AA aggravated PAN‐induced oxidative stress and apoptosis in cultured podocytes.

**FIGURE 8:**
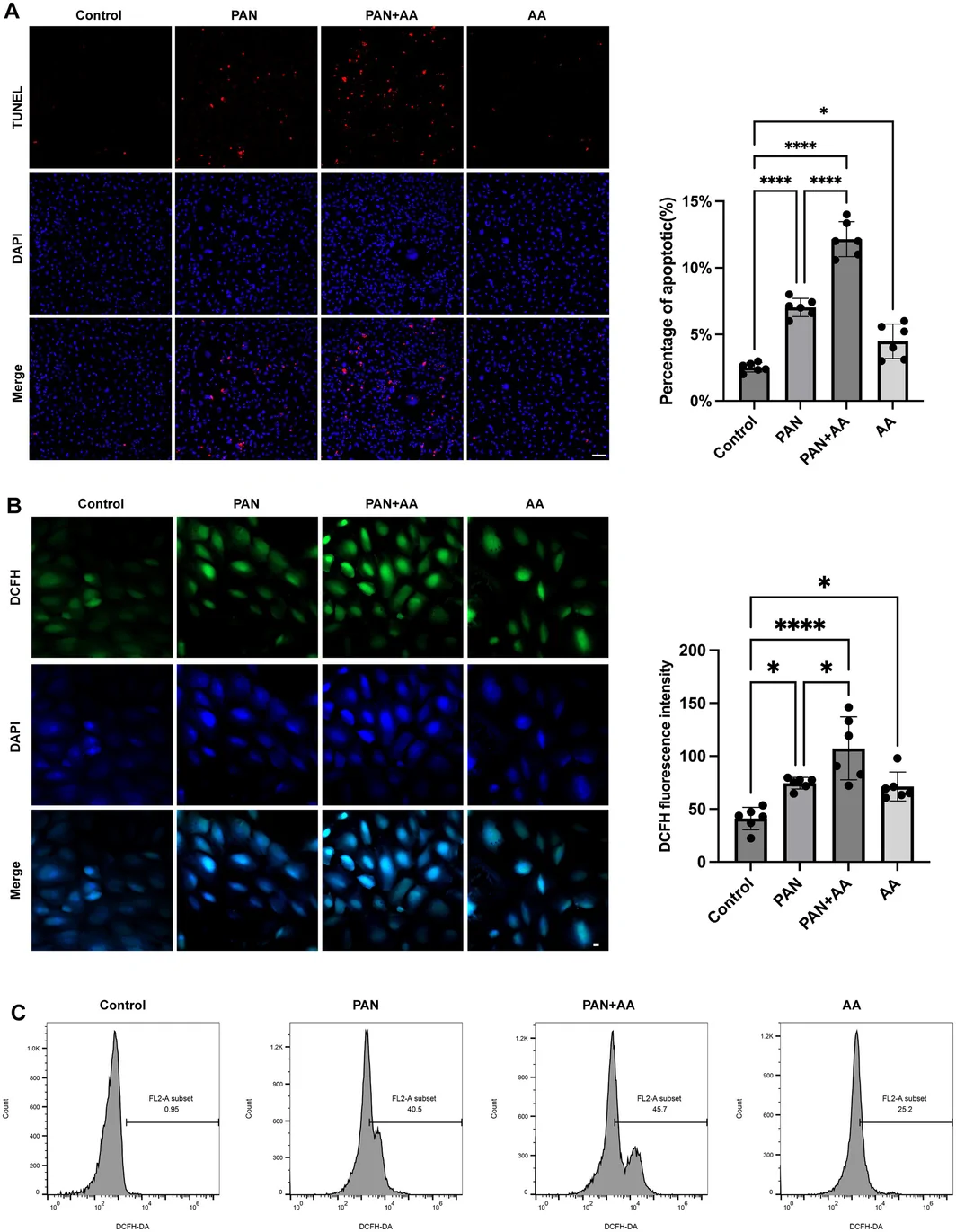
AA aggravated PAN‐induced oxidative stress and apoptosis in podocytes. Control podocytes were treated with PBS. PAN‐treated podocytes received 250 μM PAN for 24 h. PAN+AA‐treated podocytes received PAN (250 μM) and AA (5 × 10^−7^ M) for 24 h. AA‐treated podocytes received AA (5 × 10^−7^ M) for 24 h. (A) Representative TUNEL staining images and quantification of apoptotic podocytes. TUNEL‐positive cells are shown in red and nuclei in blue (*n* = 6). Scale bar, 50 μm. (B) Representative fluorescence microscopy images of DCFH‐DA‐stained podocytes and quantification of DCF fluorescence intensity. DCF fluorescence is shown in green and nuclei in blue (*n* = 6). Scale bar, 20 μm. (C) Representative flow‐cytometry histograms showing DCF flurescence in DCFH‐DA‐stained podocytes from the Control, PAN, PAN+AA, and AA groups. Data are expressed as the mean ± SD and analysed by one‐way ANOVA with Bonferroni correction. *p < 0.05, ****p < 0.0001.

### 3.5 PKM2 Activation Attenuated PAN+AA‐Induced Podocyte Injury In Vitro

We next tested whether pharmacological activation of PKM2 could attenuate PAN+AA‐induced podocyte injury. TEPP‐46 treatment increased F‐actin mean intensity and restored a more organized actin cytoskeleton in PAN+AA‐treated podocytes (Figure 9A). TUNEL staining showed that TEPP‐46 reduced the proportion of apoptotic podocytes after PAN+AA exposure (Figure 9B). TEPP‐46 also increased PKM2 expression and reduced phospho‐PKM2 abundance, as shown by immunofluorescence staining and Western blotting (Figure 9C–F). Consistently, PK activity, lactate production, pyruvate production and ATP levels were increased after TEPP‐46 treatment (Figure 9M). These findings indicate that PKM2 activation partially restored PK‐associated metabolic activity in PAN+AA‐treated podocytes.

**FIGURE 9:**
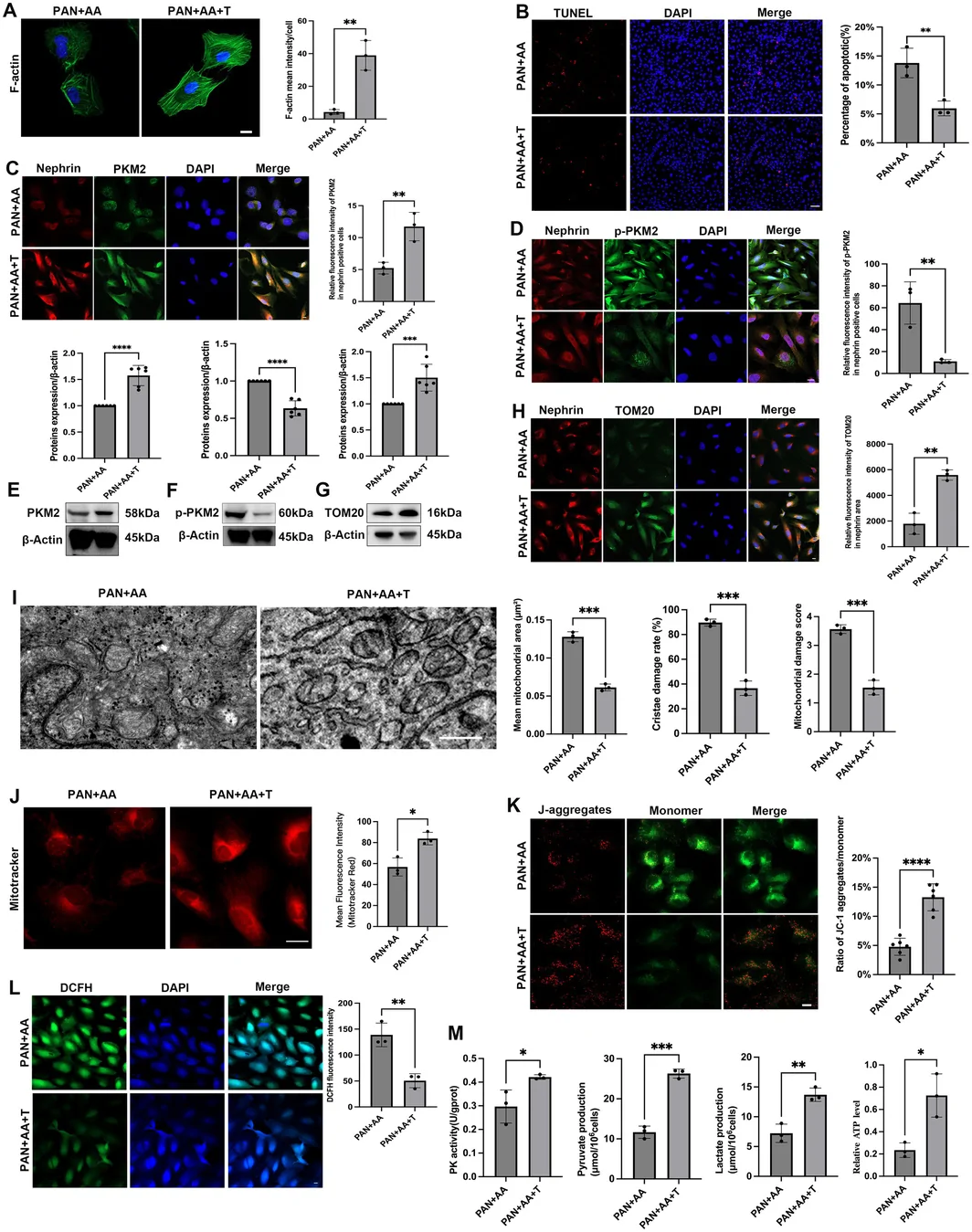
PKM2 Activation attenuated PAN + AA‐induced podocyte injury and mitochondrial dysfunction in vitro. PAN+AA‐treated podocytes were exposed to PAN (250 μM) and AA (5 × 10^−7^ M) for 24 h. PAN+AA+TEPP‐46‐treated podocytes were exposed to TEPP‐46 (10 μM) under PAN+AA conditions. T denotes TEPP‐46 in the figure. (A) Representative phalloidin staining images of F‐Actin and quantification of F‐Actin mean intensity per cell (*n* = 3). Scale bar, 20 μm. (B) Representative TUNEL staining images and quantification of apoptotic podocytes (*n* = 3). Scale bar, 50 μm. (C, D) Representative immunofluorescence images and quantification of PKM2 and phospho‐PKM2. Nephrin is shown in red, PKM2 or phospho‐PKM2 in green, and DAPI in blue (*n* = 3). Scale bar, 20 μm. (E) Western blot analysis and quantification of PKM2 protein expression (*n* = 6). (F) Western blot analysis and quantification of phospho‐PKM2 (p‐PKM2) protein expression (*n* = 6). (G) Western blot analysis and quantification of TOM20 protein expression (*n* = 6). (H) Representative immunofluorescence images and quantification of TOM20 (*n* = 3). Scale bar, 20 μm. (I) Transmission electron microscopy images and quantification of mean mitochondrial area, cristae‐damage rate and mitochondrial damage score (*n* = 3). Scale bar, 2.0 μm. (J) MitoTracker staining and quantification of mitochondrial fluorescence intensity (*n* = 3). Scale bar, 20 μm. (K) JC‐1 staining and quantification of mitochondrial membrane potential (*n* = 3). Scale bar, 20 μm. (L) Representative DCFH‐DA staining images and quantification of ROS levels (*n* = 3). Scale bar, 20 μm. (M) PK activity, lactate production, pyruvate production and ATP levels (*n* = 3). Data are expressed as mean ± SD. Statistical significance was determined by unpaired Student's *t*‐test. *p < 0.05, **p < 0.01, ***p < 0.001, ****p < 0.0001.

We then assessed whether TEPP‐46 also affected mitochondrial injury. TEPP‐46 increased TOM20 expression, as shown by immunofluorescence staining and Western blotting (Figure 9G,H). Transmission electron microscopy showed that TEPP‐46 reduced mitochondrial swelling, cristae damage, and the mitochondrial damage score in PAN+AA‐treated podocytes (Figure 9I). MitoTracker and JC‐1 staining further showed improved mitochondrial signal intensity and increased mitochondrial membrane potential after TEPP‐46 treatment (Figure 9J,K). DCFH‐DA staining showed lower ROS levels in the PAN+AA+TEPP‐46 group than in the PAN+AA group (Figure 9L). Together, these results indicate that PKM2 activation partially alleviated PAN+AA‐induced cytoskeletal injury, apoptosis, mitochondrial damage, and oxidative stress in podocytes.

## 4 Discussion

This study identifies AA as a metabolic and mitochondrial stress amplifier in PAN‐induced podocyte injury. Rather than modelling clinical relapse directly, the PAN+AA condition allowed us to test whether elevated AA can worsen established podocyte injury. Across rat kidneys and cultured human podocytes, AA exposure was associated with reduced PKM abundance, increased inhibitory PKM2 Tyr105 phosphorylation, lower PK activity, mitochondrial dysfunction, oxidative stress and apoptosis. These findings support a model in which AA amplifies PAN‐induced podocyte injury in association with PKM2‐associated metabolic dysfunction and mitochondrial stress.

Infection is a major clinical trigger of MCD relapse. AA is released during tissue injury and inflammatory activation, and excessive AA has been linked to podocyte damage. Previous work from our group showed that AA activates PKA, promotes c‐Abl activation and nephrin phosphorylation, and induces actin cytoskeletal remodelling in podocytes [24]. Other studies have reported that sPLA2IB‐PLA2R signalling promotes podocyte apoptosis through ERK1/2 and cPLA2α activation, accompanied by increased podocyte AA content [25]. These observations support a role for AA‐related lipid signalling in podocyte injury. In the present study, PAN+AA‐treated rats developed more severe proteinuria, nephrin loss and foot‐process injury than PAN‐treated rats. These findings suggest that AA can amplify PAN‐induced glomerular injury, although this model should be interpreted as AA‐exacerbated injury rather than a complete clinical relapse model.

Podocytes require coordinated energy metabolism to preserve cytoskeletal organization and filtration‐barrier integrity. Mitochondrial oxidative metabolism and glycolysis both contribute to ATP availability and stress adaptation in podocytes [26, 27, 28, 29, 30]. Mitochondrial dysfunction can reduce ATP production, increase ROS generation, and disrupt signalling pathways that maintain podocyte structure [31, 32, 33, 34, 35]. Consistent with this framework, AA aggravated mitochondrial swelling, cristae disruption, TOM20 loss, membrane‐potential reduction, and mitochondrial ROS accumulation under PAN‐induced conditions. These mitochondrial changes were accompanied by increased cellular oxidative stress and apoptosis. Thus, AA appears to shift PAN‐injured podocytes towards a more vulnerable bioenergetic and redox state.

PKM2 may provide a link between altered glycolytic metabolism and podocyte injury. In this study, PAN+AA reduced total PKM2 abundance but increased phospho‐PKM2 levels. This pattern suggests that AA affects both PKM2 expression and phosphorylation status. Because PKM2 activity depends on its abundance, oligomeric state, and post‐translational modification, increased phospho‐PKM2 should not be interpreted simply as increased glycolytic activity. Indeed, PK activity, pyruvate, lactate, and ATP were reduced after PAN+AA exposure. TEPP‐46 partially reversed these changes and increased PK activity and glycolysis‐related metabolites. These findings support the interpretation that AA is associated with impaired PKM‐linked metabolic activity in injured podocytes. However, they do not by themselves prove a direct reduction in glycolytic flux or mitochondrial respiration.

PKM2 function should be interpreted in the context of its abundance, oligomeric state, enzymatic activity and phosphorylation status [36]. Consistently, recent evidence in diabetic nephropathy showed that podocyte SIRPα reduction promotes PKM2 Tyr105 phosphorylation and nuclear translocation, leading to NF‐κB activation, oxidative stress and impaired aerobic glycolysis. Tetrameric PKM2 has higher pyruvate kinase activity and supports the conversion of phosphoenolpyruvate to pyruvate, whereas less active PKM2 states reduce terminal glycolytic output [37, 38]. TEPP‐46 has been shown to stabilize the active tetrameric state of PKM2 [39], and Tyr105 phosphorylation has been reported to inhibit PKM2 activity by disrupting formation of the active tetrameric state [40, 41]. In podocyte‐related kidney injury, pharmacological activation of PKM2 has also been reported to improve metabolic and mitochondrial dysfunction, supporting the biological plausibility of PKM2 activation as a protective mechanism [21, 42]. Increased phospho‐PKM2 Tyr105 in PAN+AA‐treated podocytes is best interpreted as a marker of PKM2 dysfunction rather than beneficial PKM2 activation. This interpretation is consistent with the TEPP‐46 rescue experiment, in which pharmacological PKM2 activation partially restored PK activity, pyruvate, lactate, ATP, mitochondrial readouts and podocyte injury markers. However, because ECAR/OCR, isotope‐tracing flux analysis and podocyte‐specific PKM2 activity assays were not performed, these findings should be interpreted as evidence of altered glycolysis‐related endpoints and PKM2‐associated dysfunction, rather than definitive proof of reduced glycolytic flux or oxidative phosphorylation.

These findings should be considered together with our previous work on AA‐induced podocyte injury. We previously reported that excessive AA promotes actin remodelling and podocyte injury through a PKA‐c‐Abl‐dependent pathway involving nephrin phosphorylation [24]. That mechanism mainly explains how AA activates kinase‐dependent slit‐diaphragm and cytoskeletal signalling. In contrast, the present study identifies a PKM2‐associated metabolic axis in which PAN+AA reduced PKM2 expression, increased PKM2 Tyr105 phosphorylation, decreased PK activity, lowered pyruvate, lactate and ATP levels, and aggravated mitochondrial injury, ROS accumulation and podocyte apoptosis. We therefore propose that AA aggravates podocyte injury through at least two convergent mechanisms: a PKA‐c‐Abl‐nephrin axis that affects nephrin signalling and actin cytoskeletal remodelling, and a PKM2‐associated metabolic axis that impairs energy metabolism and mitochondrial homeostasis (Figure 10). These pathways may interact, because actin remodelling requires sufficient ATP supply and mitochondrial ROS can further destabilize nephrin and cytoskeletal organization. However, the present data do not establish a strict sequential relationship between the two pathways. Future studies combining PKM2 gain‐ or loss‐of‐function with PKA or c‐Abl inhibition will be required to determine whether these events are upstream, downstream or parallel responses to AA‐associated stress.

**FIGURE 10:**
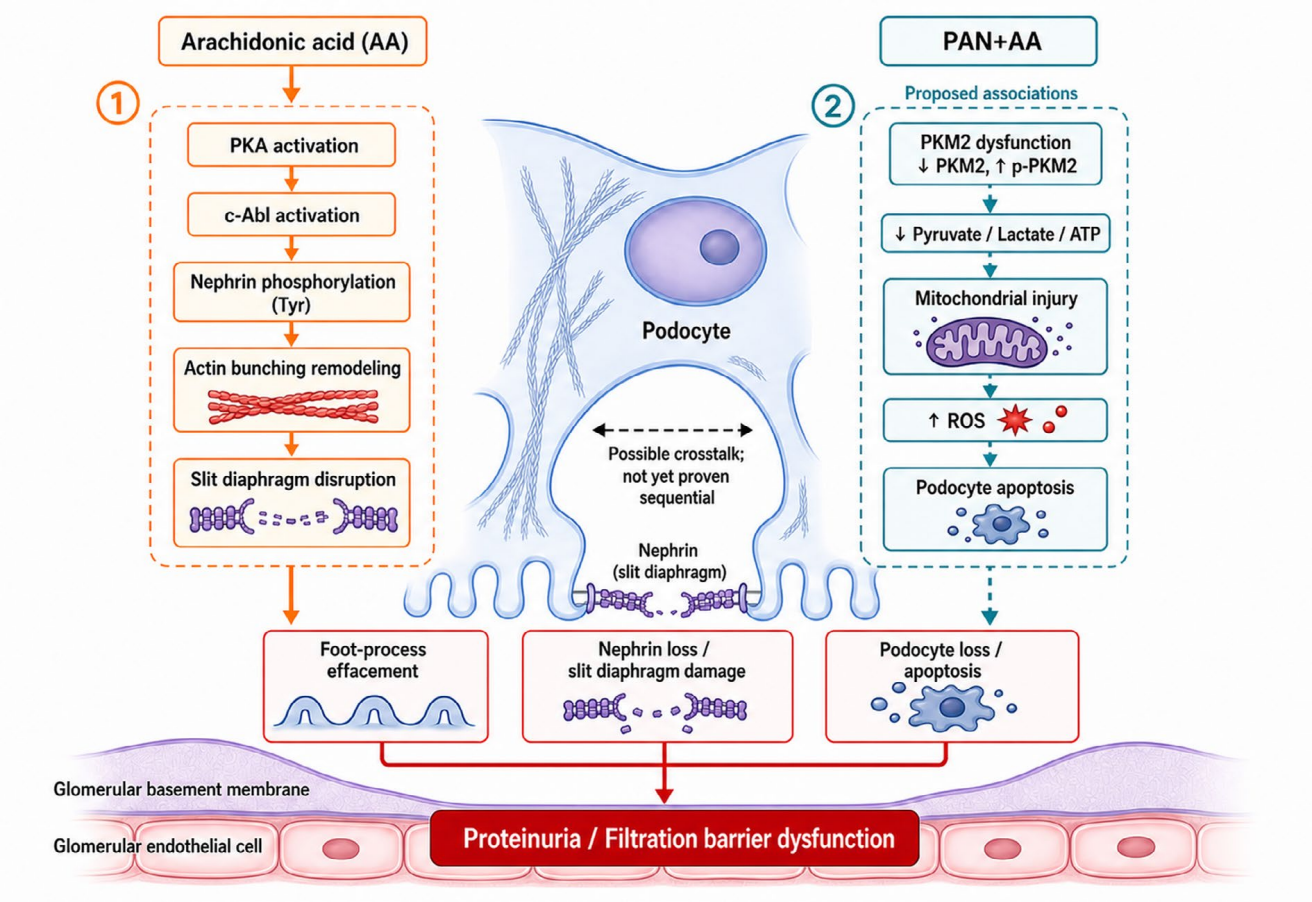
Proposed working model of AA‐associated aggravation of PAN‐induced podocyte injury. Based on our previous study, AA may activate the PKA‐c‐Abl‐nephrin axis and contribute to nephrin phosphorylation and actin cytoskeletal remodelling. In the present study, PAN+AA was associated with reduced PKM2 abundance, increased PKM2 Tyr105 phosphorylation, decreased PK activity, lower pyruvate/lactate/ATP levels, mitochondrial injury, ROS accumulation and apoptosis. These cytoskeletal and metabolic stress pathways may converge to aggravate podocyte injury and proteinuria. Potential crosstalk between these pathways is proposed, but the sequence and causal relationship remain to be experimentally validated.

This study has several limitations. First, the PAN+AA model does not fully reproduce infection‐triggered MCD relapse, because it lacks a true infectious stimulus and does not demonstrate remission followed by relapse. Second, although AA exposure was designed to model an inflammatory lipid signal, more precise lipidomic analysis would be needed to distinguish free AA, esterified AA, and downstream eicosanoid metabolites in serum and kidney tissue. Because AA is a substrate for cyclooxygenase‐derived eicosanoid production, AA may also influence injured podocytes through COX2/PGE2‐related pathways. However, COX2 expression and PGE2 production were not examined in the present study. Future studies are needed to determine whether COX2/PGE2 signalling contributes to AA‐exacerbated PAN‐induced podocyte injury. In contrast to omega‐6 AA, omega‐3 PUFA/FFAR4 signalling has recently been reported to protect against glomerular podocyte injury as well as renal ageing and CKD‐related tubular injury, highlighting the divergent biological effects of different PUFA subclasses in kidney disease [43, 44]. Third, glycolytic flux and mitochondrial respiration were not directly measured by ECAR/OCR assays, and the relative contribution of glycolysis and oxidative phosphorylation to podocyte ATP production remains unresolved. Fourth, PK activity was measured, but the specific contribution of PKM2 activity and the functional consequences of PKM2 phosphorylation require further study. Fifth, TEPP‐46 rescue experiments were performed in vitro, and in vivo PKM2 activation experiments are needed to assess therapeutic relevance.

In conclusion, AA aggravated PAN‐induced podocyte injury and was associated with impaired PKM2‐linked metabolic homeostasis, mitochondrial dysfunction, oxidative stress and apoptosis. PKM2 activation partially restored metabolic and mitochondrial readouts and attenuated podocyte injury in vitro. These findings support further testing of PKM2‐targeted metabolic intervention in AA‐associated podocyte injury, including in vivo rescue studies and models that better recapitulate infection‐associated relapse.

## Author Contributions

**Kun Nie:** writing – original draft, data curation. **Xiaowei Gao:** methodology, data curation. **Minggang Liu:** investigation, formal analysis. **Danyu You:** methodology, investigation. **Mengjie Weng:** methodology, validation. **Tingting Zheng:** methodology, validation. **Jiaqun Lin:** formal analysis. **Siyi Rao:** formal analysis. **Yongjie Zhuo:** visualization, investigation. **Jianxin Wan:** funding acquisition, project administration, supervision, resources, writing – review and editing. **Liyan Yang:** funding acquisition, visualization, writing – review and editing, project administration, resources, supervision.

## Funding

The study was funded by the National Natural Science Foundation of China (Nos. 82470733 and 82100740), and the Fujian Provincial Natural Science Foundation Youth Innovation Project (No. 2021J05154).

## Ethics Statement

All animal experiments were approved by the Institutional Animal Care and Use Committee of Fujian Medical University (Approval No. IACUC FJMU 2022‐0636).

## Consent

All authors of the manuscript have read and agreed to its content and are accountable for all aspects of the accuracy and integrity of the manuscript in accordance with ICMJE criteria.

## Conflicts of Interest

The authors declare no conflicts of interest.

## Data Availability

The data that support the findings of this study are available on request from the corresponding author. The data are not publicly available due to privacy or ethical restrictions.
